# Exercise intervention modulates food reward in obesity: efficacy, mechanisms, and prospects

**DOI:** 10.3389/fnut.2026.1895299

**Published:** 2026-08-24

**Authors:** Dongfei Liu, Yucheng Jiang, Zhanfeng Wang, Xiaohe Mo

**Affiliations:** 1 School of Physical Education, Yunnan Normal University, Kunming, China; 2 School of Physical Education, Shihezi University, Shihezi, China; 3 Department of Scientific Research, Henan Sport University, Zhengzhou, China; 4 School of Physical Education, Yunnan Minzu University, Kunming, China

**Keywords:** exercise, food reward, intervention efficacy, mechanisms, obesity

## Abstract

Aberrant food reward processing is a key contributor to the development and maintenance of obesity, and exercise intervention has been proposed as a feasible way to correct this maladaptive eating pattern. Nevertheless, inconsistent research findings mean its real efficacy and internal regulatory mechanisms remain controversial. This work adopted a systematic review design following standardized literature screening and bias evaluation procedures to sort out all qualified controlled trials focusing on exercise-induced changes in food reward among obese individuals, then synthesized existing evidence and conducted subgroup analyses to explore multiple potential moderators. Our synthesis demonstrated that exercise is effective at modulating aberrant food reward, with its outcomes jointly shaped by exercise protocols, personal traits and study design. Three interrelated regulatory axes were identified: (i) the cognitive-neural pathway that tunes mesolimbic dopamine, opioid and endocannabinoid signaling as well as prefrontal and hippocampal function; (ii) the physiological-endocrine pathway that balances gut hormones and microbiota while mitigating central insulin and leptin resistance and chronic inflammation; (iii) the psychological-behavioral pathway that strengthens inhibitory control, reduces food attentional bias and improves emotional regulation. Noticeable limitations including high outcome heterogeneity, insufficient causal evidence and the absence of personalized exercise schemes still restrict current research progress. We recommend future research to combine multi-dimensional reward measurements, multimodal omics and causal inference to unpack core mechanisms, and build phenotype-matched exercise regimens to advance obesity intervention from population-based strategies to individualized precision treatment.

## 1 Introduction

Obesity has become a major global public health challenge. According to the World Health Organization, in 2022, the number of children and adolescents aged 5–19 years with obesity exceeded 160 million worldwide, and the prevalence of obesity in this age group increased from 2% in 1990 to 8% in 2022. During the same period, the number of adults with obesity surpassed 890 million, with approximately 16% of adults aged 18 years and older living with obesity, and the global prevalence of adult obesity more than doubled compared with 1990 (1). Among the contributing factors, aberrant eating behavior, as a core pathogenic factor in the development and maintenance of obesity, can substantially exacerbate obesity risk through multiple pathways, including disruption of physiological homeostasis and heightened neural activation (2). Therefore, shifting the focus of obesity prevention and treatment from mere weight control to the correction of aberrant eating behaviors is key to moving the prevention and control gateway upstream and transforming management strategies toward a prevention-oriented and precision intervention paradigm.

The core feature of aberrant eating behavior in individuals with obesity lies in maladaptive food reward processing, manifested as heightened reward sensitivity to food cues, with its underlying mechanisms involving multisystem dysregulation across cognitive-neural, physiological-endocrine, and psychological-behavioral domains. Exercise intervention, as a non-pharmacological approach that integrates both psychological modulation and physiological enhancement, holds promise for correcting this aberrant behavior through the synergistic modulation of multisystem functions. However, current research findings on the effects of exercise on food reward remain markedly controversial, with different exercise regimens producing varying and even contradictory effects on food reward behaviors (3–6). This complex relationship not only engenders uncertainty regarding the efficacy of exercise interventions but also impedes the elucidation of the underlying mechanisms.

In light of this, through a systematic review and critical appraisal of the empirical evidence on the effects of exercise on food reward processing, this review aims to elucidate the modulatory effects of exercise interventions on food reward, to delineate the potential neural, physiological, and psychological mechanisms, and to clarify current research limitations and future directions. We seek to deepen the theoretical understanding of the mechanisms underlying food reward processing in individuals with obesity and to provide an empirical basis and theoretical reference for developing exercise-based weight management strategies in practice.

## 2 Methods

### 2.1 Literature search strategy

This study strictly followed the PRISMA 2020 (Preferred Reporting Items for Systematic Reviews and Meta-Analyses) guidelines. A combination of subject headings and free-text terms was used to systematically search eight Chinese and English databases. The Chinese databases included CNKI, VIP, Wanfang, and SinoMed; the English databases included Web of Science, PubMed, Embase, and the Cochrane Library. In addition, a supplementary search was conducted by manually screening the reference lists of the included studies to minimize the risk of overlooking relevant studies. The search covered the period from the inception of each database to May 15, 2026.

**Figure 1:**
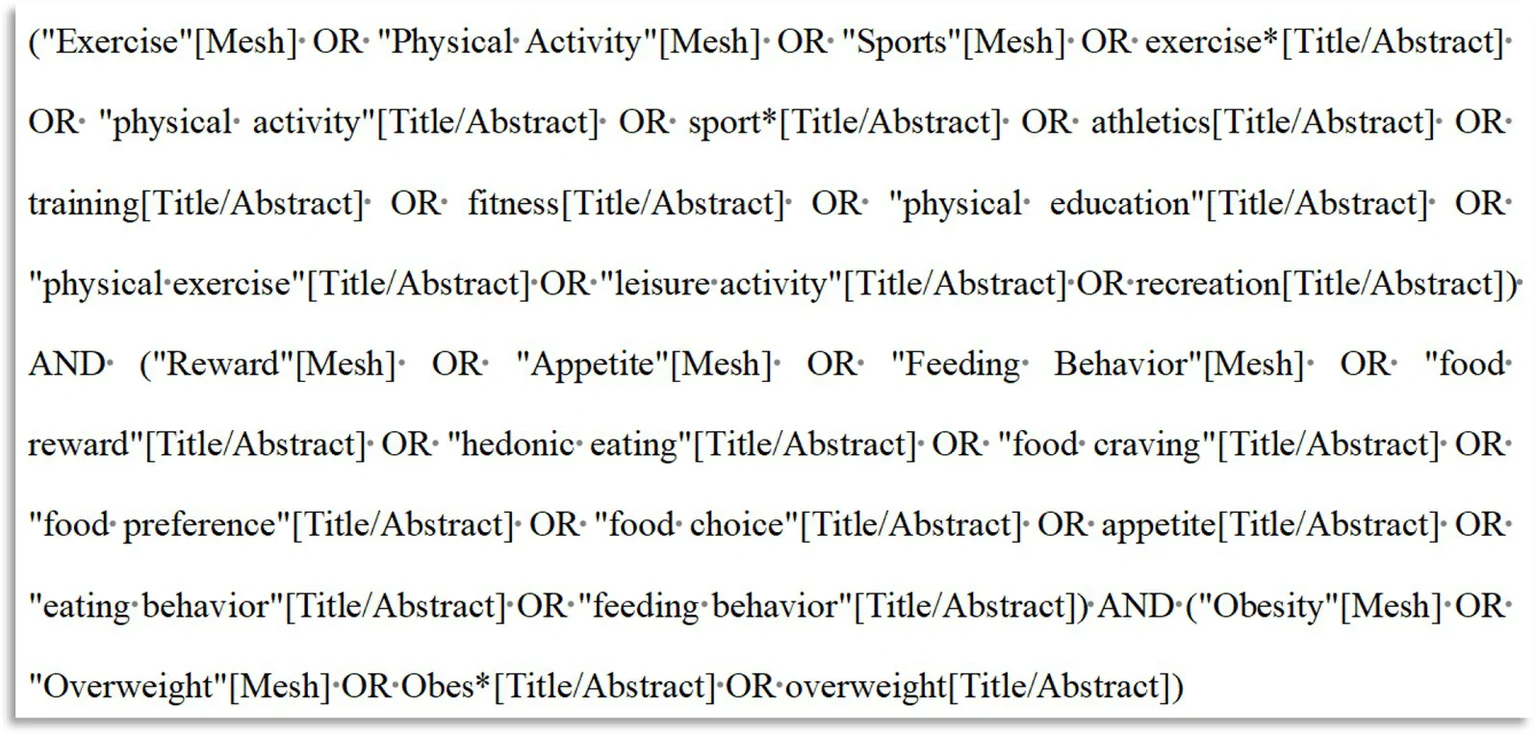
Search strategy for PubMed.

The search terms were structured around three core dimensions: “exercise intervention,” “food reward,” and “obesity.” The search terms for exercise intervention were sport OR athletics OR exercise OR training OR fitness OR physical exercise OR physical activity OR physical education OR leisure activity OR recreation. The search terms for food reward were reward OR food reward OR hedonic eating OR food craving OR food preference OR food choice OR appetite OR eating behavior OR feeding behavior. The search terms for obesity were overweight OR obesity OR obese. Terms within each dimension were combined with the Boolean operator OR, and the three dimensions were combined with AND. The search strategy was adapted to the syntactic conventions of each database. Taking PubMed as an example, the complete search string is as follows Figure 1:

### 2.2 Inclusion and exclusion criteria

Inclusion criteria: (1) Population: Individuals with a clear diagnosis of overweight or obesity based on internationally recognized BMI criteria. (2) Intervention: Any form of single or structured exercise intervention program. (3) Comparator: No exercise intervention (e.g., usual care, health education, waitlist) or an alternative exercise intervention differing in intensity or type from the intervention group. (4) Outcomes: Reporting of at least one quantitative assessment measure related to food reward. (5) Study design: Randomized controlled trials or non-randomized controlled trials.

Exclusion criteria: (1) Qualitative studies, reviews, meta-analyses, conference abstracts, editorials, commentaries, and case reports. (2) Animal experiments, *in vitro* cell experiments, and other non-human experimental studies (such studies are cited only as supporting evidence in the mechanistic discussion and are not included in the formal effectiveness analysis). (3) Duplicate publications of the same study data (only the version with the most complete data or the earliest publication date is included). (4) Studies for which the full text is unavailable or data reporting is incomplete, and the information remains unobtainable after contacting the authors. (5) Non-Chinese or non-English language publications.

### 2.3 Study selection and data extraction

Two uniformly trained reviewers independently performed the study selection according to the inclusion and exclusion criteria. The specific process was as follows: First, bibliographic records retrieved from each database were imported into EndNote X9 reference management software for automatic deduplication, supplemented by manual verification. Second, titles and abstracts were screened to exclude obviously irrelevant studies, and the number of excluded records was recorded. Finally, full texts were obtained for further screening, reasons for exclusion were documented, and eligible studies were ultimately included. Disagreements during the screening process were resolved by discussion; if consensus could not be reached, a third reviewer arbitrated.

Prior to formal data extraction, a standardized data extraction form was developed based on the PICOS framework of this study, with reference to the data extraction specifications in the Cochrane Handbook for Systematic Reviews of Interventions and the core outcome indicators of this study. After the initial draft of the form was completed, two reviewers independently conducted a pilot extraction on three sample studies. Consistency assessment was performed for the categorical items in the form, and the inter-rater agreement yielded a Cohen’s Kappa > 0.80, indicating good consistency. Based on the pilot extraction results, variable definitions, field formats, and value assignment rules were optimized and refined, and the form was finalized after review by the entire research team. All data were independently extracted by two reviewers and then cross-checked, with final verification by a third reviewer. When key information was missing or unclearly reported, the corresponding author of the original study was contacted whenever possible to obtain supplementary data.

The extracted information included: (1) Population: total sample size, sample sizes of the intervention and control groups, participant age (mean ± standard deviation or age range), BMI (mean ± standard deviation or z-score); (2) Intervention: type of exercise intervention, total intervention duration, frequency, session duration, exercise intensity; (3) Comparator: specific content of the control condition; (4) Outcomes: quantitative measures related to food reward (e.g., explicit liking, explicit wanting, implicit wanting, and relative preference as assessed by the Leeds Food Preference Questionnaire); (5) Study design: study type (e.g., randomized controlled trial, non-randomized controlled trial), time points of food reward assessment (e.g., pre-meal/post-meal), risk of bias assessment results; (6) Other information: first author, year of publication, country where the study was conducted, etc.

### 2.4 Risk of Bias assessment

Based on the study design of the included literature, corresponding internationally standardized tools were used to assess the risk of bias. For randomized controlled trials, the Cochrane Risk of Bias tool (RoB 1.0) was employed, evaluating seven domains: random sequence generation, allocation concealment, blinding of participants and personnel, blinding of outcome assessment, incomplete outcome data, selective reporting, and other sources of bias. Each domain was rated as “low risk,” “unclear risk,” or “high risk.” For non-randomized intervention studies, the Risk Of Bias In Non-randomized Studies – of Interventions (ROBINS-I) tool was applied, assessing seven domains: confounding, selection of participants, classification of interventions, deviations from intended interventions, missing data, measurement of outcomes, and selective reporting. Each domain was rated as “low risk,” “moderate risk,” “serious risk,” or “critical risk.” The overall risk of bias was determined according to the ROBINS-I criteria: an overall “low risk” rating was assigned if all domains were low risk; an overall “moderate risk” rating was assigned if all domains were “low risk” or “moderate risk”; an overall “serious risk” rating was assigned if at least one domain was “serious risk” and no domain was “critical risk”; and an overall “critical risk” rating was assigned if any domain was “critical risk.” All risk of bias assessments were performed independently by two reviewers. Prior to the assessment, reviewers received uniform training to ensure consistent understanding of the criteria. During the assessment, each reviewer independently reviewed the full texts, evaluated each domain, and recorded the judgments. After completion, results were cross-checked. Any disagreements were resolved through discussion or, when necessary, by arbitration with a third reviewer to reach consensus.

## 3 Results

### 3.1 Study selection process

**Figure 2:**
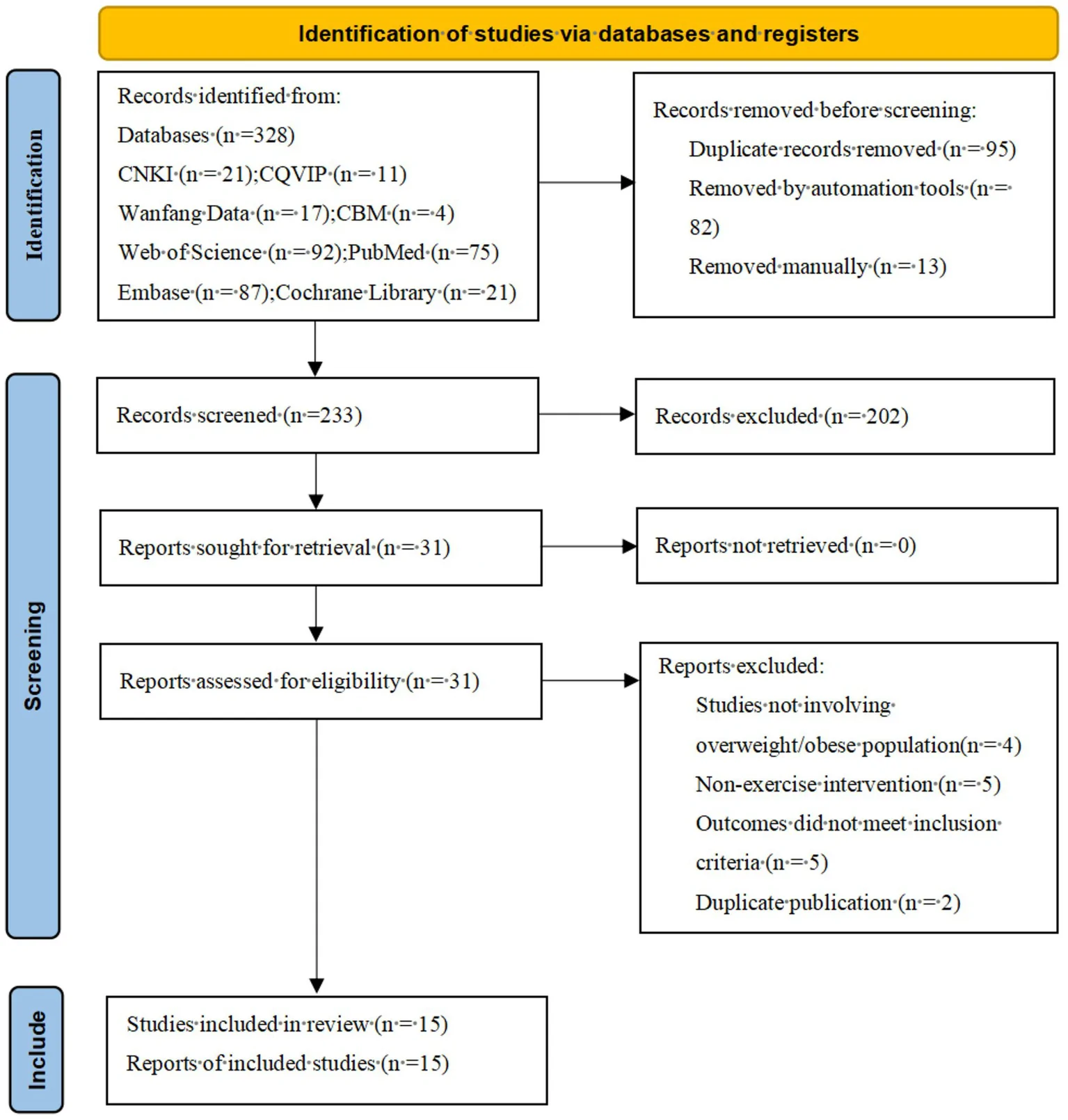
PRISMA flow diagram of the study selection process.

The initial search yielded 328 records; after screening and exclusion, 15 studies were ultimately included (5–19). The study flow diagram is shown in Figure 2.

### 3.2 Basic characteristics of the included literature

After screening, 15 publications were included (5–19), comprising 25 intervention groups that reported the independent effects of exercise interventions on food reward-related outcomes in individuals with obesity. The included studies were published between 2012 and 2024 and were conducted across multiple countries, including the United States, the United Kingdom, France, Norway, and Australia. Study designs included both randomized controlled trials and non-randomized controlled trials. The sample sizes of the intervention groups ranged from 12 to 120 participants. Exercise intervention protocols encompassed both acute and chronic exercise. The intervention modalities included aerobic exercise and interval training, with intervention durations ranging from 4 to 24 weeks, session durations of 10–60 min, and frequencies ranging from once per week to more than five times per week. Exercise intensity varied from moderate to high. The time points for food reward assessment ranged from pre-meal to several hours post-meal, and also encompassed multiple time windows from immediately post-exercise to several hours post-exercise. The primary tools for assessing food reward included the LFPQ, the PFS, and fMRI to measure participants’ neural responses to food cues. The basic characteristics of the included studies are shown in Table 1.

**Table 1:** Characteristics of included studies.

Study	Country	Age, BMI (kg/m^2^)	*n* (women)		Control	Exercise intervention program						Assessment		Intervention outcomes
			CG	IG		Intervention	Type	Period (wks)	Session (min)	Freq (wk^−1^)	Intensity	Timing	Measures	
Ross et al. (5)	USA	50.76 ± 10.38 31.19 ± 4.41	75(52)		NR	AE	2	12	NR	1	moderate intensity	NR	PFS	Food reward sensitivity ↓
Beaulieu et al. (6)	UK	CG:41.4 ± 10.7, 31.4 ± 3.7 IG: 43.2 ± 7.5, 30.6 ± 3.8	15 (9)	46 (30)	No exercise control	AE	2	12	NR	5	70% HRmax	NR	LFPQ	Explicit liking for high-fat foods – Explicit wanting for high-fat foods NR Implicit wanting for high-fat foods ↓ Relative preference for high-fat foods NR
Miguet et al. (7)	France	13.3 ± 0.9 2.3 ± 0.2 (BMIz)	33 (21)	33 (21)	No exercise control	HIIT	1	NR	15	NR	70–90% HRmax	PreM (Ex30)/ PostM0 (Ex30+)	LFPQ	PreM (Ex30): all outcomes – PostM0 (Ex30+): Explicit liking for high-fat and sweet foods – Explicit wanting for high-fat and sweet foods – Implicit wanting for high-fat and sweet foods ↓ Relative preference for high-fat and sweet foods ↓
Fillon et al. (8)	France	12.8 ± 1.4 2.2 ± 0.4 (BMIz)	17 (8)	17 (8)	No exercise control	AE (Exercise pre-meal group)	1	NR	30	NR	65% VO2peak	PreM (Ex0)/ PostM0 (Ex60)	LFPQ	PreM (Ex0): all outcomes – PostM0 (Ex60): Explicit liking for high-fat foods ↓; Explicit liking for sweet foods – Explicit wanting for high-fat and sweet foods – Implicit wanting for high-fat and sweet foods – Relative preference for high-fat and sweet foods NR
Fillon et al. (8)	France	12.8 ± 1.4 2.2 ± 0.4 (BMIz)	17 (8)	17 (8)	No exercise control	AE (Exercise post-meal group)	1	NR	30	NR	65% VO2peak	PostM0 (Ex60)	LFPQ	Explicit liking for high-fat foods ↓; Explicit liking for sweet foods – Explicit wanting for high-fat and sweet foods – Implicit wanting for high-fat and sweet foods – Relative preference for high-fat and sweet foods NR
Pelissier et al. (9)	France	13 ± 1 35.7 ± 4.1 98.8 ± 0.7 (BMI percentile)	17 (11)	17 (11)	No exercise control	AE	1	NR	38	NR	65% VO2peak	PreM	LFPQ	Explicit liking for high-fat foods –; Explicit liking for sweet foods ↓ Explicit wanting for high-fat and sweet foods – Implicit wanting for high-fat and sweet foods – Relative preference for high-fat and sweet foods –
Siroux et al. (10)	France	13.1 ± 1.3 4.48 ± 0.25 (BMIz)	13 (8)	13 (8)	No exercise control	AE	1	NR	30	NR	65% VO₂peak	PreM (Ex45)/ PostM0 (Ex45+)/ PostM6h (Ex7.5 h)	LFPQ	Explicit liking for high-fat and sweet foods – Explicit wanting for high-fat and sweet foods – Implicit wanting for high-fat and sweet foods – Relative preference for high-fat and sweet foods –
Martins et al. (11)	Norway	33.4 ± 10.0 32.3 ± 2.7	12 (7)	12 (7)	No exercise control	AE	1	NR	27	NR	70% HRmax	PreM (Ex0)	LFPQ	Explicit liking for high-fat foods – Explicit wanting for high-fat foods – Implicit wanting for high-fat foods – Relative preference for high-fat foods –
Martins et al. (11)	Norway	33.4 ± 10.0 32.3 ± 2.7	12 (7)	12 (7)	No exercise control	HIIT	1	NR	18	NR	85–90% HRmax	PreM (Ex0)	LFPQ	Explicit liking for high-fat foods – Explicit wanting for high-fat foods – Implicit wanting for high-fat foods – Relative preference for high-fat foods –
Martins et al. (11)	Norway	33.4 ± 10.0 32.3 ± 2.7	12 (7)	12 (7)	No exercise control	HIIT	1	NR	9	NR	85–90% HRmax	PreM (Ex0)	LFPQ	Explicit liking for high-fat foods – Explicit wanting for high-fat foods NR Implicit wanting for high-fat foods – Relative preference for high-fat foods –
Fillon et al. (12)	France	13.1 ± 1.4 2.3 ± 0.3 (BMIz)	15 (9)	15 (9)	No exercise control	AE (Ex60 pre-meal group)	1	NR	30	NR	65% VO2peak	PreM (Ex45)/ PostM0 (Ex60+)	LFPQ	Explicit liking for high-fat and sweet foods – Explicit wanting for high-fat and sweet foods – Implicit wanting for high-fat and sweet foods – Relative preference for high-fat and sweet foods –
Fillon et al. (12)	France	13.1 ± 1.4 2.3 ± 0.3 (BMIz)	15 (9)	15 (9)	No exercise control	AE (EX180 pre-meal group)	1	NR	30	NR	65% VO_2_peak	PreM (Ex165)/ PostM0 (Ex180+)	LFPQ	Explicit liking for high-fat and sweet foods – Explicit wanting for high-fat and sweet foods – Implicit wanting for high-fat and sweet foods – Relative preference for high-fat and sweet foods –
Fillon et al. (13)	France	12.7 ± 1.3 2.2 ± 0.4 (BMIz)	18 (6)	18 (6)	No exercise control	AE (Ex90 pre-meal group)	1	NR	30	NR	65% VO_2_peak	PreM (Ex15)/ PostM0 (Ex45)/ PostM6h15 (Ex6h45)	LFPQ	Explicit liking for high-fat foods – Explicit wanting for high-fat foods NR Implicit wanting for high-fat foods– Relative preference for high-fat foods NR
Fillon et al. (13)	France	12.7 ± 1.3 2.2 ± 0.4 (BMIz)	18 (6)	18 (6)	No exercise control	AE (Ex90 pre-meal group)	1	NR	30	NR	65% VO_2_peak	PreM (Ex75)/ PostM15 (Ex105)/ PostM5h15 (Ex6h45)	LFPQ	PreM (Ex75)/PostM15 (Ex105): all outcomes – PostM5h15 (Ex6h45): Explicit liking for high-fat foods↓ Explicit wanting for high-fat foods NR Implicit wanting for high-fat foods – Relative preference for high-fat foods NR
Cornier et al. (14)	USA	38.2 ± 9.5 33.3 ± 4.3	12(5)		NR	AE	2	24	15–60	5	60–75% VO₂max	NR	fMRI	Neural responses to food cues in bilateral parietal lobes, left insula, etc. ↓
Hopkins et al. (15)	UK	18–55 30.8 ± 3.5(women) 30.5 ± 4.7(men)	46(30)		NR	AE	2	12	NR	5	70% HRmax	NR	LFPQ	Explicit liking for high-fat foods – Explicit wanting for high-fat foods NR Implicit wanting for high-fat foods – Relative preference for high-fat foods NR
Martins et al. (16)	Norway	34.4 ± 8.8 33.3 ± 2.9	14		NR	AE	2	12	32	3	70% HRmax	NR	LFPQ	Explicit liking for high-fat and sweet foods – Explicit wanting for high-fat and sweet foods NR Implicit wanting for high-fat and sweet foods – Relative preference for high-fat and sweet foods –
Martins et al. (16)	Norway	34.4 ± 8.8 33.3 ± 2.9	16		NR	HIIT	2	12	20	3	85–90% HRmax	NR	LFPQ	Explicit liking for high-fat and sweet foods – Explicit wanting for high-fat and sweet foods NR Implicit wanting for high-fat and sweet foods – Relative preference for high-fat and sweet foods –
Martins et al. (16)	Norway	34.4 ± 8.8 33.3 ± 2.9	16		NR	HIIT	2	12	10	3	85–90% HRmax	NR	LFPQ	Explicit liking for high-fat and sweet foods – Explicit wanting for high-fat and sweet foods NR Implicit wanting for high-fat and sweet foods – Relative preference for high-fat and sweet foods –
Thivel et al. (17)	France	12–16 31.8 ± 3.8	12(6)		NR	AE (Concentric exercise group)	2	12	NR	3	NR	NR	LFPQ	Explicit liking for high-fat and sweet foods – Explicit wanting for high-fat and sweet foods – Implicit wanting for high-fat and sweet foods – Relative preference for high-fat and sweet foods –
Thivel et al. (17)	France	12–16 34.8 ± 5.5	12(6)		NR	AE (Eccentric exercise group)	2	12	NR	3	NR	NR	LFPQ	Explicit liking for high-fat and sweet foods – Explicit wanting for high-fat and sweet foods – Implicit wanting for high-fat foods –; Implicit wanting for sweet foods ↓ Relative preference for high-fat foods ↑; Relative preference for sweet foods ↓
Martin et al. (18)	USA	48.9 ± 11.4 31.5 ± 4.7	61 (45)	59 (43)	NR	AE (Low-dose group)	2	24	NR	≥5	65–85%VO₂peak	NR	FPQ	Explicit liking for high-fat and sweet foods NR Explicit wanting for high-fat and sweet foods NR Implicit wanting for high-fat and sweet foods NR Relative preference for high-fat and sweet foods –
Martin et al. (18)	USA	48.9 ± 11.4 31.5 ± 4.7	61 (45)	59 (43)	NR	AE (High-dose group)	2	24	NR	≥5	65–85%VO₂peak	NR	FPQ	Explicit liking for high-fat and sweet foods NR Explicit wanting for high-fat and sweet foods NR Implicit wanting for high-fat and sweet foods NR Relative preference for high-fat and sweet foods ↓
Alkahtani et al. (19)	Australia	29 ± 3.7 30.7 ± 3.4	10 (0)	10 (0)	NR	MIIT	2	4	37.5	3	45 ± 20% VO₂peak	NR	LFPQ	Explicit liking for high-fat foods ↑ Explicit wanting for high-fat and sweet foods NR Implicit wanting for high-fat and sweet foods – Relative preference for high-fat and sweet foods NR
Alkahtani et al. (19)	Australia	29 ± 3.7 30.7 ± 3.4	10 (0)	10 (0)	NR	HIIT	2	4	18.7	3	90% VO₂peak	NR	LFPQ	Explicit liking for high-fat foods ↓ Explicit wanting for high-fat and sweet foods NR Implicit wanting for high-fat and sweet foods – Relative preference for high-fat and sweet foods NR

BMI, body mass index (unit: kg/m^2^, plain numerical values without parentheses for adults and adolescents without z-score data); BMIz, BMI z-score (values labelled with (BMIz) in parentheses represent standardized BMI for adolescents); CG, control group; IG, intervention group; n, subgroup total sample size (value in parentheses denotes the number of female participants within this group); AE, aerobic training; HIIT, high-intensity interval training; MIIT, moderate-intensity interval training; 1, acute exercise; 2, chronic exercise; wks, weeks; min, minutes; wk^−1^, per week; Freq, weekly exercise frequency; PreM, pre-meal assessment; PostM0, immediate post-meal assessment; PostM15, assessment 15 min after meal; PostM30, assessment 30 min after meal; PostM5h15, assessment 5 h 15 min after meal; PostM6h, assessment approximately 6 h after meal; PostM6h15, assessment 6 h 15 min after meal; Ex0, immediately after exercise; ExXX, XX minutes after exercise; Ex7.5 h, 7.5 h after exercise; Ex6h45, 6 h 45 min after exercise.↑, significant increase vs. baseline or control group; ↓, significant decrease vs. baseline or control group; −, no significant difference vs. baseline or control group; NR,not Reported, not separately reported in the original studies; PFS, Power of Food Scale; fMRI, functional magnetic resonance imaging; LFPQ, Leeds Food Preference Questionnaire; FPQ, Food Preference Questionnaire.

### 3.3 Risk of Bias assessment results

Of the 15 included studies, 11 were randomized controlled trials and were assessed for risk of bias using the Cochrane Risk of Bias tool (RoB 1.0). The remaining four studies were non-randomized controlled trials and were evaluated using the ROBINS-I tool. Overall, the risk of bias in the 15 included studies stemmed primarily from two sources: in the randomized controlled trials, the key limitations were insufficient reporting of randomization details and the inherent challenges of blinding; in the non-randomized intervention studies, the key limitation was confounding bias arising from single-arm designs or baseline imbalance between groups. The detailed risk-of-bias assessments for each domain are presented in Tables 2, 3.

**Table 2:** Risk of bias assessment for randomized controlled trials.

Study	Random sequence generation	Allocation concealment	Blinding of participants and personnel	Blinding of outcome assessment	Incomplete outcome data	Selective reporting	Other sources of bias
Miguet et al. (7)	Unclear	Unclear	High	Low	Low	Low	Low
Fillon et al. (8)	Unclear	Unclear	High	Low	Low	Low	Low
Pelissier et al. (9)	Unclear	Unclear	High	Low	Low	Low	Low
Siroux et al. (10)	Unclear	Unclear	High	Low	Unclear	Low	Low
Martins et al. (11)	Unclear	Unclear	High	Low	Low	Low	Low
Fillon et al. (12)	Unclear	Unclear	High	Low	Low	Low	Low
Fillon et al. (13)	Unclear	Unclear	High	Low	Low	Low	Low
Martins et al. (16)	Unclear	Unclear	High	Low	Unclear	Low	Unclear
Thivel et al. (17)	Unclear	Unclear	High	Low	Unclear	Low	Unclear
Martin et al. (18)	Low	Low	High	Low	Unclear	Low	Low
Alkahtani et al. (19)	Unclear	Unclear	High	Low	Low	Low	Low

Risk categories: Low, High, Unclear.

**Table 3:** Risk of bias assessment for non-randomized studies.

Study	Confounding	Selection of participants	Classification of interventions	Deviations from intended interventions	Missing data	Measurement of outcomes	Selective reporting	Overall risk of bias
Ross et al. (5)	Moderate	Low	Low	Moderate	Low	Low	Low	Moderate
Beaulieu et al. (6)	Moderate	Low	Low	Moderate	Low	Low	Low	Moderate
Cornier et al. (14)	Moderate	Low	Low	Moderate	Low	Low	Moderate	Moderate
Hopkins et al. (15)	Moderate	Low	Low	Low	Moderate	Low	Moderate	Moderate

Risk categories: Low, Moderate, Serious, Critical.

### 3.4 Effects of exercise intervention on food reward processing in individuals with obesity

From the 15 included studies, a total of 25 intervention arms reporting the independent effects of exercise interventions on food reward behavior in individuals with obesity were extracted. Among these, 10 intervention arms demonstrated an inhibitory effect on at least one food reward dimension, with no dimension showing enhancement; 13 intervention arms observed no significant changes in any food reward dimension; 1 intervention arm reported a mixed effect (a significant increase in one dimension and a significant decrease in another); and 1 intervention arm reported an enhancing effect on food reward.

With respect to individual food reward dimensions, 21 intervention arms reported the effects of exercise on explicit liking. Of these, 5 showed a significant decrease in explicit liking after exercise, 1 showed a significant increase in explicit liking for high-fat foods, and 15 observed no significant effect. Eleven intervention arms reported on explicit wanting, all of which observed no significant effect. Twenty-one intervention arms reported on implicit wanting; among them, 3 showed a significant decrease in implicit wanting after exercise, and 18 observed no significant effect. Fifteen intervention arms reported on relative preference; of these, 2 showed a significant decrease in relative preference after exercise, 1 reported a mixed effect (a significant decrease in relative preference for sweet foods and a significant increase in relative preference for high-fat foods), and 12 observed no significant effect.

With respect to exercise type, 12 intervention arms reported the effects of acute exercise on food reward. Of these, 5 showed an inhibitory effect on at least one food reward dimension, and 7 observed no significant effect. Thirteen intervention arms reported the effects of chronic exercise on food reward. Of these, 5 showed an inhibitory effect on at least one food reward dimension, 1 reported a mixed effect (a significant increase in one dimension and a significant decrease in another), 1 reported an enhancing effect on food reward, and 6 observed no significant effect.

### 3.5 Moderating effects of different factors

#### 3.5.1 Moderating effects of exercise type

The included studies primarily comprised two types of exercise: interval training and aerobic exercise.

For acute exercise, 3 intervention arms involved interval training, of which 1 observed an inhibitory effect; 9 intervention arms involved aerobic exercise, of which 4 observed an inhibitory effect.

For chronic exercise, 4 intervention arms involved interval training, of which 1 observed an inhibitory effect; 9 intervention arms involved aerobic exercise, of which 4 observed an inhibitory effect.

#### 3.5.2 Moderating effects of exercise intervention duration

As acute exercise does not involve an intervention duration, this subgroup analysis was conducted only for chronic exercise. The intervention durations in the included studies mainly fell into three categories: 4 weeks, 12 weeks, and 24 weeks. The 4-week subgroup included 2 intervention arms, of which 1 observed an inhibitory effect. The 12-week subgroup included 8 intervention arms, of which 2 observed an inhibitory effect. The 24-week subgroup included 3 intervention arms, of which 2 observed an inhibitory effect.

#### 3.5.3 Moderating effects of exercise intervention frequency

As acute exercise does not involve an intervention frequency, this subgroup analysis was conducted only for chronic exercise. The intervention frequencies in the included studies were mainly divided into two levels: ≤3 times/week and ≥5 times/week. The ≤3 times/week subgroup included 8 intervention arms, of which 2 observed an inhibitory effect. The ≥5 times/week subgroup included 5 intervention arms, of which 3 observed an inhibitory effect.

#### 3.5.4 Moderating effects of exercise intervention intensity

The included studies were categorized by exercise intensity into high intensity (≥70% HRmax) and moderate intensity (≤65% VO₂peak, ≤70% HRmax).

For acute exercise, the high-intensity subgroup included 3 intervention arms, of which 1 observed an inhibitory effect; the moderate-intensity subgroup included 9 intervention arms, of which 3 observed an inhibitory effect.

For chronic exercise, the high-intensity subgroup included 3 intervention arms, of which 1 observed an inhibitory effect; the moderate-intensity subgroup included 6 intervention arms, of which 3 observed an inhibitory effect.

#### 3.5.5 Moderating effects of single exercise session duration

The included studies were categorized by session duration into short duration (≤20 min) and moderate duration (20–40 min).

For acute exercise, the short-duration subgroup included 3 intervention arms, of which 1 observed an inhibitory effect; the moderate-duration subgroup included 9 intervention arms, of which 4 observed an inhibitory effect.

For chronic exercise, the short-duration subgroup included 3 intervention arms, of which 1 observed an inhibitory effect; the moderate-duration subgroup included 2 intervention arms, neither of which observed an inhibitory effect.

#### 3.5.6 Moderating effects of assessment timing

As the included chronic exercise studies did not report information on testing timing (pre-meal/post-meal), the time interval from the end of eating to testing, or the time interval from the end of exercise to testing, it was not feasible to conduct a relevant time-effect analysis. Therefore, the exploration of assessment timing in this study was limited to acute exercise.

Regarding testing timing (pre-meal/post-meal), a total of 11 intervention arms conducted food reward tests pre-meal after exercise, of which 1 observed an inhibitory effect; a total of 8 intervention arms conducted food reward tests post-meal after exercise, of which 4 observed an inhibitory effect.

Regarding the time interval between the end of eating and the food reward test, the testing time points of the included intervention arms were concentrated in two periods: within 30 min post-meal and over 5 h post-meal. Tests within 30 min post-meal covered a total of 7 time points, of which 3 observed an inhibitory effect; tests over 5 h post-meal involved a total of 3 time points, of which 1 observed an inhibitory effect.

Regarding the time interval between the end of exercise and the food reward test, the testing time points of the included intervention arms were divided into four subgroups. The ≤30 min post-exercise subgroup included 6 time points, none of which observed an inhibitory effect; the >30 and ≤60 min post-exercise subgroup included 5 time points, of which 2 observed an inhibitory effect; the >60 and ≤120 min post-exercise subgroup included 3 time points, none of which observed an inhibitory effect; the >120 min post-exercise subgroup included 5 time points, of which 1 observed an inhibitory effect.

#### 3.5.7 Moderating effects of age

The included studies were categorized by participant age into children and adolescents (<18 years) and adults (≥18 years).

For acute exercise, the children and adolescents subgroup included 9 intervention arms, of which 5 observed an inhibitory effect; the adults subgroup included 3 intervention arms, none of which observed an inhibitory effect.

For chronic exercise, the children and adolescents subgroup included 2 intervention arms, neither of which observed an inhibitory effect; the adults subgroup included 11 intervention arms, of which 5 observed an inhibitory effect.

#### 3.5.8 Moderating effects of sex

As none of the included original studies reported sex-stratified effect sizes or sex-by-exercise intervention interaction terms, it was not possible to assess the moderating effect of sex based on individual-level data. Therefore, this study conducted an exploratory subgroup analysis using the proportion of females in the sample, with a pre-specified threshold of 50% to categorize studies into a low female proportion group (<50% female) and a high female proportion group (≥50% female).

For acute exercise, the low female proportion subgroup included 4 intervention arms, of which 3 observed an inhibitory effect; the high female proportion subgroup included 8 intervention arms, of which 2 observed an inhibitory effect.

For chronic exercise, the low female proportion subgroup included 3 intervention arms, of which 2 observed an inhibitory effect; the high female proportion subgroup included 7 intervention arms, of which 3 observed an inhibitory effect.

## 4 Discussion

### 4.1 Mechanisms underlying the effects of exercise intervention on food reward processing in obesity

The accumulating evidence reviewed above supports the efficacy of exercise interventions in modulating food reward processing in individuals with obesity. Beyond this phenomenological level, however, a critical question concerns the mechanisms through which these effects are achieved; the following sections synthesize current evidence across multiple levels of analysis to outline a tentative mechanistic framework. Before examining the specific mechanisms, it is important to clarify the nature and hierarchy of the evidence discussed below. The mechanistic pathways proposed in this review are derived from evidence spanning multiple levels of inquiry, including rodent models of diet-induced obesity, human neuroimaging studies, and observational data from exercise interventions. While animal studies provide valuable insights into molecular and circuit-level mechanisms that are not feasible in humans, their translation to clinical practice requires caution. Throughout this section, findings from animal experiments are presented as candidate mechanisms rather than established causal pathways in humans, and we explicitly indicate where evidence is preliminary or requires further validation. Where human data are available, they are given greater interpretive weight, and we note the distinction between correlational neuroimaging findings and causal evidence.

#### 4.1.1 Cognitive-neural mechanisms

##### 4.1.1.1 Exercise modulates dopaminergic and glutamatergic pathways in the nucleus accumbens to ameliorate food reward function

The dopaminergic pathway projecting from the VTA to the NAc and the glutamatergic pathways projecting from the PFC and amygdala to the NAc have been identified in animal models as the core neural circuitry regulating food reward (20). In rodent models, chronic high-fat diet consumption induces compensatory suppression of the reward system, characterized by deficient DA function and excessive glutamatergic plasticity, thereby driving overeating to compensate for the reward deficit. Regular exercise, however, can normalize reward dysfunction by modulating these pathways, evidence that provides animal-level support for understanding the regulation of human food reward (21). Specifically, in the DAergic pathway, exercise has been shown to not only directly elevate DA levels in the NAc of obese mice, but also promote the expression of tyrosine hydroxylase (TH) in the substantia nigra pars compacta–dorsal striatum pathway and the VTA–NAc pathway, indirectly increasing striatal DA availability and effectively facilitating neurotransmission in these circuits (20). Concurrently, exercise upregulates D2R levels in the NAc of obese mice, ameliorating the downregulation of D2R expression and the aberrant VTA–NAc pathway activity linked to enhanced DA–D1R binding induced by long-term high-fat diet (21).

In the glutamatergic pathway, studies in obese rodents have shown that the number of α-amino-3-hydroxy-5-methyl-4-isoxazolepropionic acid receptors (AMPARs) lacking the GluA2 subunit on the cell membrane of NAc neurons is significantly increased. The high Ca^2+^ permeability of these receptors directly enhances glutamatergic synaptic transmission efficacy, thereby lowering the reward threshold (20). Exercise intervention not only directly reduces the number of AMPAR- and calcium-permeable AMPA receptor (CP-AMPAR)-positive cells on the NAc cell membrane surface, but also increases the phosphorylation level of the GluA2 subunit within AMPARs and its occupancy at synapses. The incorporation of the GluA2 subunit renders AMPARs impermeable to Ca^2+^, thereby attenuating the excessive encoding of highly rewarding foods by the NAc circuitry and reducing pathological eating motivation. Crucially, animal studies further reveal that the DAergic function restored by exercise can suppress the synaptic trafficking of CP-AMPARs, demonstrating an interactive mechanism through which DA signaling negatively regulates glutamatergic synaptic plasticity (22, 23).

These findings from animal models provide mechanistically plausible explanations for the exercise-induced changes in reward-related brain activity observed in human neuroimaging studies. However, it is important to recognize that the molecular and synaptic mechanisms identified in rodents represent candidate pathways rather than being definitively established in humans. Direct evidence linking these specific molecular changes to alterations in human reward behavior is currently lacking, and the translational gap between animal models and human obesity remains to be systematically addressed.

##### 4.1.1.2 Exercise modulates the opioid and endocannabinoid systems to ameliorate hedonic eating behavior

The EOS plays a central role in regulating hedonic eating and motivational behavior. Pathological obesity is frequently accompanied by a global reduction in brain MOR density, which results in an enhanced anticipatory reward response upon stimulation by food cues (24). In human intervention studies, aerobic exercise has been shown to induce upregulation of MOR expression, with this effect widely distributed across reward-related brain regions including the ventral striatum, medial prefrontal cortex, anterior cingulate cortex, and insula (25).

The ECS also plays an important role in hedonic eating behavior, and the obese state is often associated with dysregulated ECS signaling in key brain regions. Currently, human evidence on exercise-induced ECS modulation remains limited; however, findings from rodent studies provide valuable mechanistic insights into how exercise may regulate food reward processing. In the PFC, chronic exercise significantly upregulates CB1 receptor expression in rodents, enhancing local endocannabinoid signaling tone. This molecular change is hypothesized to strengthen top-down cognitive control, which may explain the improved food-related decision-making observed in human exercise trials (26). In the hippocampus, enhanced CB1 receptor signaling is proposed as a key molecular substrate for neuroplasticity related to dietary hedonic memory. In rodent models, chronic exercise specifically enhances hippocampal ECS function, evidenced by elevated expression of the CB1 receptor gene (Cnr1) alongside widespread upregulation of genes encoding synthetic and degradative enzymes. These changes collectively promote the turnover and signaling efficacy of hippocampal endocannabinoids (27). In the hypothalamus, exercise downregulates CB1 receptor expression in rodents, which attenuates CB1-mediated inhibition of GABAergic inputs to proopiomelanocortin (POMC) neurons in the arcuate nucleus. This in turn enhances anorexigenic signaling via *α*-melanocyte-stimulating hormone and reduces excitatory drive to orexigenic neurons in the lateral hypothalamic area (27, 28).

Notably, an interesting observation from animal studies is that long-term exercise and palatable diet induce similar ECS regulatory patterns in key brain regions—upregulation of CB1 receptor expression in the prefrontal cortex and hippocampus and downregulation of CB1 expression in the hypothalamus—yet, against this background, exercise attenuates weight gain. This phenomenon tentatively suggests that exercise may not simply substitute for food reward signals; rather, through neural adaptive mechanisms similar to those engaged by hedonic eating, it may form functional compensation in circuits involved in the integration of metabolism and reward, potentially contributing to a positive intervention against unhealthy eating behavior. However, this interpretation remains speculative, as it is derived from rodent data, and the extent to which these ECS-mediated mechanisms operate in humans—particularly in the context of exercise interventions for obesity—has yet to be determined.

##### 4.1.1.3 Exercise modulates the function of cognition-related brain regions to optimize food reward evaluation

In overweight and obese populations, exercise can reshape the function of reward circuitry by modulating the activity and functional connectivity of brain regions involved in food reward processing. The underlying mechanisms involve multi-level regulation of reward valuation, interoceptive integration, and memory processing. First, regular exercise reduces the reactivity of the OFC to high-calorie food cues, thereby optimizing the balance between cognitive control and impulsive responding during reward-based decision-making (29). Second, studies have found that higher levels of physical activity are significantly correlated with reduced activation of the insula in response to images of high-calorie foods, suggesting that exercise may attenuate acute hedonic responses to eating by modulating the integration of interoceptive signals with food hedonic value in the anterior insula (29, 30). Finally, the hippocampus, as a key brain region involved in episodic memory and contextual cue association, also plays an important role in exercise interventions. Research has shown that exercise can improve hippocampal insulin sensitivity and activate the intracellular PI3K/Akt signaling pathway, thereby increasing brain-derived neurotrophic factor (BDNF) levels and enhancing neuroplasticity. This, in turn, optimizes the encoding and retrieval of food-related episodic memories and weakens the reward responses elicited by environmental food cues (31). Taken together, these findings indicate that exercise does not act on a single brain region, but rather modulates the food reward system in an integrated manner by coordinating a broad neural network.

#### 4.1.2 Physiological-endocrine mechanisms

##### 4.1.2.1 Exercise modulates gastrointestinal hormones and the gut microbiome to regulate food reward

Dysregulation of the energy intake control pathway in individuals with obesity constitutes an important physiological basis for subsequent hedonic eating. Regular exercise can exert beneficial effects on energy balance by modulating the function of the gut–brain axis, with appetite-regulating hormones and the gut microbiome playing key roles in this process. At the gastrointestinal hormone level, research has shown that acute high-intensity exercise can significantly alter neural responses to images of high- and low-calorie foods in reward-related brain regions of individuals with obesity, a change associated with decreased concentrations of acylated ghrelin and increased release of peptide YY (PYY) (32). Furthermore, multiple studies have confirmed that exercise can reduce acylated ghrelin levels and promote increased concentrations of various anorexigenic gut peptides, such as PYY, glucagon-like peptide-1 (GLP-1), and pancreatic polypeptide, in overweight/obese individuals (7, 33, 34). Evidence indicates that these hormonal changes are associated with attenuated neurocognitive responses of reward-related brain regions to visual food cues (32).

Beyond hormonal regulation, the gut microbiome serves as another key mediator of gut-brain axis effects on food reward processing. Observational studies in humans have shown that gut microbiome dysbiosis frequently coexists with food reward system dysfunction in individuals with obesity, though a direct causal link between the two has not been established in human populations. Preclinical rodent studies have demonstrated that both high-intensity interval training and cold-water swimming can effectively ameliorate high-fat diet-induced gut dysbiosis, as evidenced by increased *α*-diversity, enrichment of beneficial bacterial taxa (e.g., Lachnospiraceae and Prevotellaceae), and enhanced microbial metabolic functions (e.g., the tricarboxylic acid cycle activity) (35, 36). This finding from rodent models provides indirect preclinical evidence for the potential pathway whereby exercise modulates food reward processing via gut microbiota remodeling. The exercise-reshaped gut microbiota may, in turn, reduce the transmission of gut-derived inflammatory factors to the central nervous system, alleviate neuroinflammation in reward-related brain regions, and consequently modulate the function of key molecules involved in DA metabolism (e.g., TH and dopamine receptors), as well as indirectly influence central food reward perception and motivation through metabolites such as short-chain fatty acids (37).

It should be noted that, although existing evidence preliminarily suggests the potential for exercise to regulate food reward through the gut microbiome, direct causal evidence linking “microbiota alterations” to “reward behavior modulation” is still lacking. Furthermore, most of the mechanistic evidence linking the gut microbiome to food reward regulation is derived from animal models; whether analogous gut–brain–reward interactions occur in humans following exercise, and whether such effects are of sufficient magnitude to influence eating behavior, remain open questions. Future studies should integrate fecal microbiota transplantation, multi-omics approaches, and brain imaging techniques to systematically validate this pathway in both animal models and human populations.

##### 4.1.2.2 Exercise alleviates chronic inflammation to restore reward circuitry function

The systemic chronic inflammation triggered by excessive adipose tissue accumulation constitutes an important pathological mechanism underlying aberrant food reward processing in individuals with obesity. Studies have demonstrated that inflammatory cytokines can disrupt the function of reward-related neural circuits through multiple pathways. Specifically, interleukin-6 (IL-6) can inhibit DA synthesis by reducing BH₄ bioactivity, while tumor necrosis factor-α (TNF-α) decreases DA release, accelerates its reuptake, and induces MOR desensitization and epigenetic suppression (38). Moreover, TNF-α also impairs AMPAR/NMDAR function at glutamatergic synapses (39). At the metabolic level, inflammatory cytokines such as TNF-α, IL-6, and interleukin-1β (IL-1β) can induce central insulin resistance and leptin resistance, thereby disrupting the integration of energy and reward signals (39, 40). Collectively, these alterations lead to attenuated reward signaling, diminished pleasure, and aberrant eating motivation. Multiple studies have shown that exercise intervention can significantly improve the inflammatory profile of overweight and obese populations, as evidenced by reductions in pro-inflammatory factors such as high-sensitivity C-reactive protein (hs-CRP), IL-6, TNF-α, and IL-1β, accompanied by enhanced secretion of anti-inflammatory cytokines such as interleukin-10 (IL-10) (41, 42). This suggests that exercise may indirectly modulate the food reward system by regulating the inflammatory state. However, direct evidence supporting the exercise–inflammation–reward modulation pathway remains lacking, and the specific molecular and neural circuit mechanisms await further investigation.

##### 4.1.2.3 Exercise ameliorates insulin and leptin signaling pathways to optimize food reward regulation

Obesity-related insulin resistance induced by long-term high-fat diet consumption leads to compensatory elevations in peripheral and central insulin levels, which in turn impair insulin signal transduction in the NAc. This impairment is specifically manifested as disrupted phosphorylation of the insulin receptor (InsR) and its substrate insulin receptor substrate 2 (IRS2), as well as reduced activation of the downstream key effector protein kinase B (Akt), thereby weakening the regulatory capacity of insulin over DA release (43). Studies have demonstrated that regular aerobic exercise can effectively reverse this signaling deficit by upregulating InsR/IRS2 expression and enhancing Akt phosphorylation in the NAc, thus restoring insulin signaling sensitivity (43, 44). The restored insulin signaling pathway can further inhibit downstream glycogen synthase kinase-3β (GSK3β) activity (43) and promote the expression of the DA synthesis rate-limiting enzyme TH in the VTA via insulin signaling (44), thereby optimizing the function of the DAergic reward pathway, reducing excessive seeking of high-fat foods, and facilitating weight control and the restoration of metabolic homeostasis.

Leptin, as a key adipokine conveying energy storage information to the central nervous system, not only regulates energy balance by stimulating the hypothalamic arcuate nucleus to induce satiety, but also participates in inhibiting food-related reward by acting on the VTA (45). The precise mechanisms through which leptin resistance develops and impacts reward circuitry have been extensively studied in animal models. In obese rodents, dysregulated adipose tissue secretion triggers hyperleptinemia and central leptin resistance, rendering leptin unable to effectively suppress the hypothalamic feeding center or constrain the excessive responsiveness of the mesolimbic system to highly rewarding foods, thereby manifesting as failed satiety signaling and enhanced reward-driven eating (46). A meta-analysis has confirmed that aerobic, resistance, and combined exercise can all significantly reduce circulating leptin levels in overweight/obese individuals (47). This effect, combined with findings from animal models, suggests that exercise may reverse central leptin resistance—not merely through passive reductions in leptin secondary to fat loss, but potentially by restoring the sensitivity of reward-related brain regions to leptin signaling. However, this mechanistic pathway is largely derived from animal studies on how leptin resistance develops and affects reward circuitry. Whether these mechanisms operate similarly in humans directly determines whether exercise-induced changes in leptin sensitivity translate into meaningful improvements in food reward behavior in individuals with obesity—a question that requires further investigation.

#### 4.1.3 Psychological-behavioral mechanisms

##### 4.1.3.1 Exercise enhances inhibitory control to reduce food reward sensitivity

Individuals with obesity often exhibit deficits in PFC-mediated inhibitory control, leading to heightened impulsive responses to highly rewarding food cues. Exercise has been shown to enhance food-related inhibitory control, thereby helping individuals with obesity overcome their elevated food reward sensitivity and reduce impulsive eating behavior. Empirical studies have demonstrated that both a single bout of high-intensity interval exercise and continuous moderate-intensity aerobic exercise can improve the inhibitory control of overweight individuals in response to high-calorie food stimuli (48). This benefit may be associated with exercise-induced increases in dlPFC blood flow and changes in neurotransmitter release (49). Further research has revealed that, following a single bout of high-intensity interval exercise, individuals not only exhibit enhanced inhibitory control but also show significantly reduced delay discounting of food rewards, with concurrent decreases in hedonic evaluation of high-energy-density foods and the tendency to select ideal portion sizes (49). This suggests that exercise-enhanced inhibitory control can effectively intervene in and modify the rapid reward evaluation and decision-making processes triggered by food cues, thereby reducing impulsive eating behavior by improving the cognitive efficacy of dietary self-regulation.

##### 4.1.3.2 Exercise modulates attentional Bias to reduce the salience of high-calorie food cues

Individuals with obesity generally exhibit an attentional approach bias toward high-calorie foods, characterized by attention being easily captured by food cues and difficulty in disengaging. This automatic cognitive processing pattern significantly increases the risk of cue-induced craving and overeating in food-rich environments. Exercise can serve as an effective intervention to reshape the attentional processing of food cues. Studies have shown that acute exercise can enhance the attention of individuals with obesity toward the health attributes of food while reducing their attentional bias toward high-calorie food cues (50, 51). Neuroelectrophysiological evidence further reveals that, following acute resistance exercise, overweight/obese individuals exhibit shortened latencies of the N2 and P3 components and reduced N2 amplitude when processing food cues. This indicates that exercise can not only accelerate the speed of attentional orientation and allocation toward food cues in overweight/obese individuals, but also reduce the automatic attentional capture by food-related stimuli (51). Such alterations in attentional processing patterns may be related to the attenuated responses of brain regions involved in reward, memory, and visual processing to high-calorie food cues, with BDNF potentially playing a key mediating role (52, 53).

##### 4.1.3.3 Exercise improves emotion regulation to alleviate emotional eating

**Figure 3:**
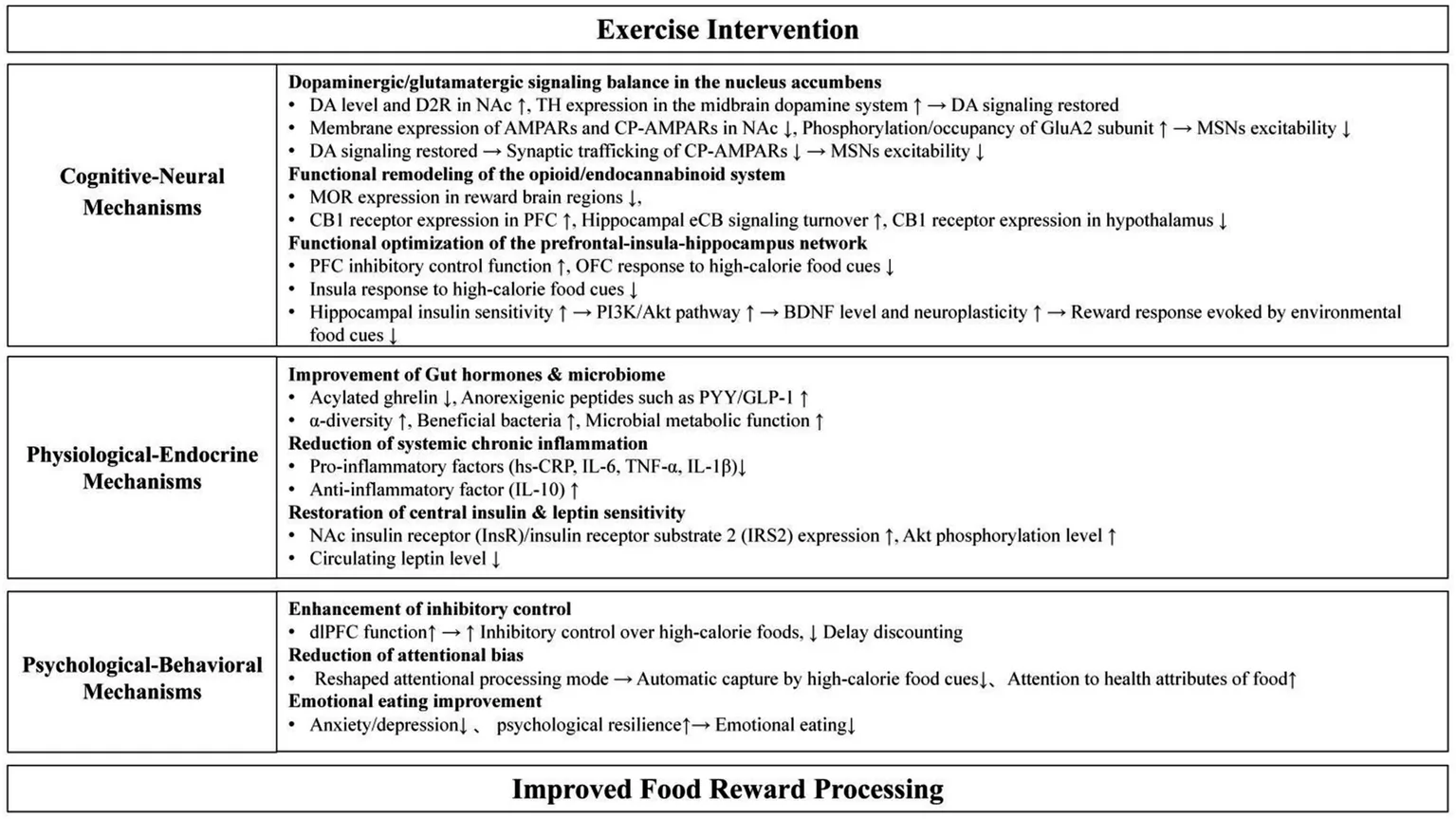
Regulatory mechanisms of exercise intervention on food reward in obesity.

Emotional eating is a key psychological-behavioral factor in the development and maintenance of obesity, essentially representing the use of highly rewarding food intake as a maladaptive strategy for coping with negative emotions. Regular exercise, through its mood-enhancing benefits, can help individuals with obesity rebuild healthy emotion regulation strategies, thereby disrupting the aberrant association between negative emotions and food reward. A large body of evidence demonstrates that exercise can effectively alleviate symptoms of anxiety and depression, enhance stress coping capacity, and promote psychological resilience—benefits that have direct implications for the improvement of emotional eating behavior in individuals with obesity. A 12-week exercise intervention significantly reduced anxiety symptoms and the consequent binge eating behavior in individuals with obesity (54). A 10-month intervention study further found that exercise not only reduced emotional eating behavior, but also decreased post-consumption food liking and attenuated reward responsivity during the transition from hunger to satiety (55). This indicates that the effects of exercise extend beyond behavioral suppression of eating to fundamentally reshape the hedonic system, weakening the reward value of food as an emotional compensator and thereby reestablishing the balance between the homeostatic and hedonic systems (see Figure 3).

### 4.2 Analysis of the moderating effects of different factors on the effects of exercise interventions on food reward

#### 4.2.1 Analysis of the moderating effects of factors related to exercise intervention protocols

Given that this study did not employ meta-regression to quantitatively analyze the moderating effects of various exercise intervention protocol-related factors on exercise-induced modulation of food reward in individuals with obesity, it instead conducted a semi-quantitative synthesis through subgroup stratification to map the effects of exercise interventions on food reward under different conditions. Therefore, the discussion in this section aims to present exploratory effect patterns and to provide a reference for future mechanistic research and trial design. The relevant results should not be directly generalized as definitive conclusions.

With respect to exercise modality, the present review found that aerobic exercise appeared to be slightly superior to interval training in both acute and chronic exercise contexts, preliminarily suggesting that the inhibitory effect of aerobic exercise on food reward may be more pronounced than that of interval training. This difference may be attributable to the distinct mechanistic pathways through which the two exercise modalities regulate the reward system. The modulation of food reward by aerobic exercise involves multi-level neural and physiological pathways. At the neural level, aerobic exercise can upregulate the expression of tyrosine hydroxylase and D2 receptors in the mesolimbic dopamine system, thereby ameliorating high-fat diet-induced abnormalities in reward signaling (20, 21), and can also reduce the reward threshold by modulating glutamatergic synaptic plasticity in the nucleus accumbens (22, 23). In addition, aerobic exercise can regulate hedonic eating behavior by modulating the region-specific expression of *μ*-opioid receptors and cannabinoid type 1 receptors (25–27). At the physiological level, aerobic exercise can reduce acylated ghrelin levels and increase the concentrations of anorexigenic hormones such as PYY and GLP-1, thereby transmitting homeostatic inhibitory signals via the gut–brain axis (7, 33, 34); it can also restore insulin signaling in the nucleus accumbens, alleviate central insulin resistance, and attenuate food reward drive from a metabolic perspective (43, 44). In contrast, the modulation of food reward by interval training may rely more heavily on cognitive control pathways. Studies have shown that acute high-intensity interval exercise can improve inhibitory control and working memory, thereby reducing hedonic evaluation by weakening the approach bias toward high-calorie foods (48). Neuroelectrophysiological evidence indicates that interval training can optimize the efficiency of prefrontal–striatal neural pathways and strengthen top-down control over eating impulses (49). In summary, aerobic exercise engages a broader range of neural and physiological targets in modulating food reward, which may partially explain its more stable and pronounced inhibitory effect compared with interval training. However, simply comparing the proportion of positive studies may be insufficient to comprehensively evaluate the relative merits of different exercise modalities. Future studies should systematically compare the differential modulatory effects of the two exercise modalities on various aspects of the reward system under isoenergetic conditions, incorporating both neuroimaging and physiological indicators.

It should be noted that the literature included in the present review predominantly focused on aerobic exercise and interval training; however, emerging evidence preliminarily suggests that resistance exercise may also act on food reward regulatory pathways. McNeil et al. compared the effects of acute aerobic and resistance exercise on food reward in healthy individuals and found that both exercise modalities reduced the relative preference for high-fat foods, but a reduction in hedonic “liking” for high-fat foods was observed only with resistance exercise (56). This finding suggests that resistance exercise may influence the hedonic evaluation of food reward through unique mechanistic pathways. From a mechanistic perspective, the modulation of food reward by resistance exercise may be more dependent on the endocrine function of skeletal muscle. Contracting skeletal muscle can release various myokines, among which irisin has been proposed as a potential molecular bridge linking physical activity to reward-related processes and motivation (57). Lactate has likewise been described as a “myokine” with inter-organ crosstalk functions, serving as a signaling molecule in appetite-regulating pathways (58). Furthermore, resistance exercise can induce rapid hormonal responses, manifested by decreased ghrelin levels and increased insulin levels, thereby modulating hedonic-related neural pathways. In terms of weight management, resistance exercise can achieve the dual benefit of increasing lean body mass while reducing fat mass, and elevate resting metabolic rate by increasing muscle mass, providing a higher “basal energy expenditure” foundation for long-term energy balance maintenance, thereby synergistically reducing body weight through both body composition and energy expenditure pathways (59). Previous studies have demonstrated a positive correlation between reward sensitivity and changes in body weight, suggesting that resistance exercise may indirectly modulate food reward sensitivity through its unique advantages in weight management (5). However, the above mechanistic framework still requires validation in populations with obesity, and future studies should incorporate resistance exercise intervention protocols to directly compare the specific modulatory effects of different exercise modalities on food reward in individuals with obesity.

With respect to exercise intervention duration, the present review found that longer-duration exercise interventions may be more conducive to suppressing food reward than shorter-duration interventions. Notably, the trajectory of this effect over the course of the intervention was not entirely linear. Alkahtani et al. found that the initial phase of an exercise intervention may temporarily enhance explicit liking for savory foods, whereas this liking level gradually declined after the intervention continued for 12 to 14 weeks (19). Riou et al. (60) further indicated that compensatory increases in appetite may emerge during the early phase of the intervention due to negative energy balance, and that food reward-related eating behavior and associated indicators do not stabilize at lower levels until the intervention is sustained over a longer period. This trend may be related to the cumulative neural adaptation effects associated with longer intervention durations. Cornier et al. (14), in a 24-week exercise intervention, found that long-term exercise effectively reduced neural activation in the insula, parietal cortex, and visual cortex in response to food cues, and that the magnitude of the reduction in insular neural response was significantly positively correlated with the reduction in body fat (17). These findings suggest that longer intervention durations may gradually reduce the neural salience of high-calorie food cues through the synergistic effects of cumulative body fat loss and neural adaptation.

With respect to exercise intervention frequency, the present review found that the proportion of studies reporting significant reductions in food reward indicators was higher among studies implementing exercise interventions ≥5 times per week than among those implementing interventions ≤3 times per week, preliminarily suggesting that a higher weekly exercise frequency may be more conducive to suppressing food reward. Panek et al. (61) found that, compared with no exercise or low-frequency exercise (1–3 days per week), high-frequency exercise (5 days per week) not only reduced the reinforcing value of high-energy-density foods but also significantly increased the reinforcing value of low-energy-density foods, suggesting that a higher exercise frequency may optimize dietary choices by reshaping the reinforcing value of foods differing in energy density. This frequency-dependent reward modulation effect may be associated with altered neural reactivity to food cues in reward-related brain regions. Relevant studies have indicated that higher levels of self-reported physical activity are associated with lower reactivity to food cues in the insula, orbitofrontal cortex, postcentral gyrus, and precuneus (62).

With respect to single exercise session duration, based on the currently available data, no stable or clear moderating trend of session duration on the effects of exercise on food reward has been observed. Existing evidence suggests that both shorter- and longer-duration exercise can induce significant changes in food reward. From a theoretical perspective, extending the duration of a single exercise session can increase total energy expenditure and induce more pronounced fluctuations in appetite-regulating hormones and central stress signals, potentially amplifying the inhibitory effect on food reward. However, a longer exercise duration also exacerbates the body‘s energy deficit, which may activate energy compensation pathways and, in turn, enhance the reward value of high-calorie foods. The superposition of these two opposing effects may attenuate any unidirectional gradient effect of session duration on food reward.

With respect to exercise intervention intensity, the overall trend across the included studies indicated that the effects of moderate- and high-intensity exercise on food reward did not exhibit a clear dose–response gradient. This may be partly attributable to heterogeneity in exercise protocols and participant populations across studies, coupled with the limited number of studies at each intensity level, which is insufficient to support definitive conclusions regarding intensity effects. Moreover, original studies that directly compared different exercise intensities within the same experimental framework also reported inconsistent results. Alkahtani et al. (19), comparing high- and moderate-intensity interval training within the same experimental design, found that explicit liking for high-fat, non-sweet foods tended to decrease after high-intensity training but increased after moderate-intensity training. Riou et al. (60) likewise reported that implicit wanting for high-fat foods was higher in the low-intensity exercise group than in the moderate-intensity group. These findings suggest that higher intensity may be more conducive to suppressing reward responses to high-fat foods. However, Martins et al. found no significant differences between different intensities in either acute or chronic exercise interventions, suggesting that intensity effects may be modulated by other factors (11, 16). Furthermore, a recent study in sedentary individuals also demonstrated no intensity-specific differences in the effects of moderate-to-vigorous versus low-intensity aerobic exercise on food reward (4). These inconsistent results suggest that the relationship between exercise intensity and reward effects may not be a simple linear one.

It should be noted that the above subgroup analyses of exercise modality, duration, frequency, session duration, and intensity are all based on a limited number of intervention arms, with restricted statistical power, making it difficult to reliably distinguish random fluctuations from true effect differences. Moreover, the included studies exhibit varying degrees of confounding among these parameters, making it challenging to independently isolate the specific moderating effect of any single parameter. Therefore, the above findings should be regarded as exploratory trends rather than definitive conclusions. Future studies should, while controlling for other exercise parameters, adopt controlled trials with graded designs to systematically compare the differential effects of each parameter on various dimensions of food reward, thereby clarifying their independent moderating effects.

Beyond the above exercise parameters, energy balance status, as a cumulative outcome of exercise interventions, may also exert an important modulatory effect on food reward. Cornier et al. demonstrated that the magnitude of neural activation in the insula in response to food cues was significantly negatively correlated with changes in body fat following exercise interventions in individuals with obesity, with greater reductions in body fat associated with stronger neural inhibition in response to high-fat food cues and lower neural food reward responses (14). Given that body fat content is an objective biomarker of long-term cumulative net energy balance, this finding preliminarily suggests an association between energy balance status and the effects of exercise interventions on food reward. Martin et al. (18), by establishing two standardized weekly exercise energy expenditure conditions (low dose: 8 kcal/kg/week; high dose: 20 kcal/kg/week), induced long-term negative energy balance of differing magnitudes. The results showed that the high-dose group achieved a larger cumulative energy deficit, accompanied by significant reductions in body fat, and exhibited significantly decreased reward preferences for high-fat and high-carbohydrate foods; in contrast, the low-dose group showed no notable body fat loss and even a slight increase in reward preferences for high-calorie foods. This long-term intervention study provides compelling evidence that the cumulative magnitude of negative energy balance modulates the exercise-induced suppression of food reward. Findings from acute exercise studies further support this conclusion. Miguet et al. (7) found that when the negative energy balance difference between the exercise and control groups was only 50 kcal, no statistically significant differences were observed in any food reward indicators; however, when the difference widened to 170 kcal, implicit wanting and relative preference for high-fat and sweet foods were significantly suppressed in the exercise group. Fillon et al. (8) similarly observed that food reward exhibited inhibitory changes as negative energy balance progressively increased. Collectively, the above evidence suggests that a dose–response relationship exists between the cumulative magnitude of negative energy balance and the downregulation of food reward, and that physiological signals driven by negative energy balance (such as leptin) may serve as an important metabolic basis linking exercise to changes in food reward.

#### 4.2.2 Analysis of the moderating effects of individual characteristics-related factors

At the individual difference level, characteristics such as sex, age, body composition, psychiatric comorbidity status, baseline physical activity level, metabolic health status, habitual dietary patterns, and exercise experience may all influence the effects of exercise interventions on food reward.

With respect to the sex characteristics of the included study populations, the present review found that the proportion of intervention arms in which an inhibitory effect was observed was higher in the low-female-proportion subgroup than in the high-female-proportion subgroup. This distribution pattern preliminarily suggests that the inhibitory effect of exercise on food reward in individuals with obesity may be relatively more pronounced in samples with a higher proportion of males, and that sex composition may play a potential moderating role. One possible explanation for this finding is that sex differences exist in the neural responses of the central reward system. Functional magnetic resonance imaging studies have demonstrated that, when exposed to high-calorie food cues, women exhibit significantly greater activation than men in reward-salience networks, including the insula, orbitofrontal cortex, and amygdala (63), and show more sustained attentional bias toward visual food stimuli with greater difficulty in attentional disengagement (64). Accordingly, it can be inferred that this heightened neural reactivity may lead to higher reward valuation of food cues in women, thereby partially counteracting the downregulatory effect of exercise on the reward system. It should be emphasized, however, that the subgroup comparisons in the present review were based on study-level sex composition rather than individual-level sex data, and thus individual-level sex differences cannot be directly inferred. Therefore, this finding should be regarded as an exploratory observation rather than a definitive conclusion.

With respect to the age characteristics of the included study populations, the present review found that the inhibitory effect of acute exercise on food reward was more pronounced in children and adolescents, whereas the inhibitory effect of chronic exercise was more pronounced in adult samples. These results suggest that the modulatory effects of exercise on food reward may exhibit differentiated response patterns depending on developmental stage and intervention duration. The underlying reason may be that the reward system responds differently to exercise stimuli across developmental stages. Adolescence represents a critical window for the remodeling of the mesolimbic dopamine system, characterized by heightened neuroplasticity, during which neurochemical fluctuations induced by a single bout of exercise are more likely to trigger immediate functional changes in reward circuitry (65, 66). In contrast, the adult reward system is relatively stable, and a single bout of exercise may be insufficient to induce detectable changes; instead, the cumulative effects of long-term training are required to gradually induce neural adaptations. It should be noted that the age subgroup stratification in the present review was limited by an uneven distribution of samples, with relatively few intervention arms in the adult acute exercise and adolescent chronic exercise subgroups. Therefore, the observed age-related differences should be regarded as exploratory trends and do not yet possess robust evidential strength.

With respect to body composition characteristics, studies have shown that regular moderate-intensity exercise can significantly reduce food reward sensitivity in overweight and obese individuals, and that reward sensitivity is positively correlated with changes in body weight (5). The neural basis of this association may involve obesity-related adaptive alterations in dopamine signaling pathways. Striatal D2-like receptors represent a core node through which the mesolimbic dopamine system regulates reward motivation, and reduced availability of these receptors may attenuate neural responsiveness to natural rewards, thereby driving compensatory eating behavior. Ribeiro et al. found that when comparing obese individuals across different obesity grades with normal-weight controls, no significant difference in striatal D2-like receptor availability was observed between the obese and control groups. However, when the analysis was restricted to patients with grade III or higher obesity, D2-like receptor availability was significantly lower in the obese group than in controls (67). A related systematic review further confirmed an inverse association between BMI and D2-like receptor availability, with lower receptor availability at higher obesity grades (68). The above evidence suggests that the differences in dopamine signaling accompanying baseline obesity severity may lead to differentiated effects of exercise interventions on reward modulation. It should be noted that, according to the World Health Organization obesity classification criteria for children, adolescents, and adults, the study populations included in the present review were predominantly concentrated within the mild obesity range, with very limited representation of severe obesity, which is insufficient to support semi-quantitative comparative analyses across different obesity grades. Therefore, the present review only provides a preliminary, qualitative discussion of the potential moderating role of body composition. Future studies should include participants across a wider spectrum of obesity grades to determine whether baseline obesity severity constitutes an independent moderating variable in the exercise–food reward relationship.

With respect to metabolic health status, obesity and metabolic diseases such as type 2 diabetes have been shown to be associated with significant alterations in food reward processing, among which insulin resistance and leptin resistance may represent important pathways influencing food reward processing and the effects of exercise interventions. With respect to leptin, Hopkins et al. found that fasting leptin was significantly positively correlated with both explicit “liking” and implicit “wanting” for high-fat foods, and that the magnitude of exercise-induced leptin reduction was significantly negatively correlated with changes in explicit “liking” for high-fat foods, suggesting that leptin may serve as an important metabolic messenger linking energy balance and food reward (15). With respect to insulin signaling, a systematic review by Qiu et al. indicated that insulin-related pathways are involved in the exercise-induced modulation of food reward, and that aberrant insulin signaling may interfere with the regulatory effects of exercise on food reward by affecting dopamine system function (69). A neuroimaging study by Adam et al. (70) demonstrated that, in overweight Hispanic girls, neural activation patterns in regions such as the insula, orbitofrontal cortex, and anterior cingulate cortex in response to food cues were significantly correlated with insulin sensitivity, suggesting that insulin resistance may alter the responsiveness of reward-related brain regions to food cues. However, the exercise intervention studies included in the present review generally lacked adequate reporting of baseline metabolic health indicators. Future studies should systematically assess participants’ metabolic health status at baseline and examine interaction effects between metabolic health status and exercise protocols in their analyses.

With respect to habitual dietary pattern characteristics, relevant studies have shown that long-term high-fat diet consumption can alter feeding behavior through the mesolimbic dopamine signaling pathway, induce anhedonia and aberrant food choice, and thereby entrench preferences for high-calorie foods (71). Beyond the chronic adaptive effects of long-term dietary patterns, macronutrient composition can also influence the activation level of the reward system. Neuroimaging studies have demonstrated that the ratio of dietary fat to carbohydrates can differentially modulate neural activity in hypothalamic homeostatic regions and reward-related brain areas (such as the insula and orbitofrontal cortex) in response to food cues (72). The above evidence suggests that habitual dietary patterns may influence the effects of exercise interventions by altering the baseline functional state of the reward system. However, the original studies included in the present review did not uniformly collect data on participants’ habitual dietary patterns, precluding quantitative analysis of the interaction between dietary patterns and exercise. The present review can therefore only provide qualitative inferences based on the available evidence, and future studies should further examine the moderating effect of this factor.

With respect to baseline physical activity level, the level of physical activity prior to enrollment may be a key individual difference variable influencing the direction and magnitude of the effects of exercise on food reward. Horner et al., by comparing food reward responses between active and inactive men in a satiated state, found that active individuals had lower overall “liking” ratings for food than inactive individuals and exhibited stronger implicit “wanting” for low-fat savory foods (73). Beaulieu et al. (74), in their systematic review, further integrated evidence from observational, acute, and chronic exercise training studies, indicating that low levels of physical activity are associated with higher “liking” and “wanting” for high-energy foods, and that acute exercise tends to reduce behavioral indicators of reward for high-energy foods in inactive individuals. These behavioral findings are further supported at the neural level: a systematic review by Dera et al. (62) confirmed that higher levels of self-reported physical activity are associated with lower neural reactivity to high-energy-density food cues in regions such as the insula, orbitofrontal cortex, postcentral gyrus, and precuneus. The above evidence suggests that baseline physical activity level not only modulates an individual’s baseline sensitivity to food reward but may also influence their reward response to exercise interventions. Inactive individuals may be more sensitive to the acute reward-suppressing effects of exercise, whereas habitually active individuals may already be at a lower baseline reward level, and thus the incremental effect of exercise may be limited. However, the studies included in the present review mostly identified sedentary populations as the study sample using the International Physical Activity Questionnaire, and no subgroup analyses or interaction tests have been conducted across gradients of baseline physical activity to examine the effects of exercise interventions. Therefore, the quantitative moderating role of baseline physical activity level on the exercise–food reward relationship could not be further explored. Future research should adopt standardized criteria for stratifying baseline physical activity levels to clarify its independent moderating effect.

With respect to exercise experience characteristics, the exercise training experience of participants prior to enrollment may be a potential moderating variable influencing the effects of exercise on food reward. Cross-sectional studies have shown that habitual exercisers and sedentary individuals differ in their neural responses to food reward, with habitual exercisers exhibiting lower neural responses to high-energy food cues. This finding suggests that exercise experience may modulate individuals’ response patterns to subsequent exercise interventions by altering the baseline sensitivity of the reward system (74). Furthermore, individuals with long-term exercise experience demonstrate superior motor control quality and neuromuscular coordination, along with significantly improved exercise economy. These adaptive physiological changes can reshape the activation patterns of dopaminergic pathways during exercise and alter the magnitude of peripheral metabolic signal release, thereby indirectly modifying the inhibitory effect of exercise on food reward. However, the exercise intervention studies included in the present review generally adopted “engaging in regular exercise for no more than 2 h per week in the past 6 months” as an inclusion criterion, meaning that all enrolled participants were exercise-naïve. This design choice precludes quantitative comparisons of reward response differences between individuals with and without exercise experience. Future studies should include participants with varying levels of exercise experience to clarify the moderating role of this variable.

With respect to psychiatric comorbidity characteristics, existing evidence suggests that depressive disorders and eating disorders may modulate the effects of exercise interventions by influencing reward processing. Individuals with depressive disorders often exhibit reward processing abnormalities, manifested as altered cognitive–behavioral responses to food-related rewards. Among these, emotional eating is one of the most prominent behavioral phenotypes in depressed populations, with approximately 30%–50% of individuals with depression displaying varying degrees of disordered eating behavior. Notably, emotional eating in depressed populations is not unidirectional: emotional overeating may be characterized by excessively heightened reward responses accompanied by inhibitory control deficits, whereas emotional undereating is characterized by blunted reward processing (75). Beyond depressive disorders, eating disorders are also closely associated with reward processing abnormalities. In eating disorders such as binge eating disorder, increased variability in reward signaling is significantly associated with BMI and disinhibited eating. A systematic review by Blanchet et al. (76) demonstrated that physical activity can reduce binge eating episodes and improve associated comorbidities through mechanisms such as influencing neurochemical changes in the reward system and reducing negative affect and anorexigenic effects. Beaulieu et al. (6) and Annesi (77) further confirmed that physical activity can effectively reduce binge eating tendencies and related emotional eating in overweight/obese populations. The above evidence suggests that exercise may indirectly modulate food reward processing by improving psychiatric comorbidity symptoms. However, the vast majority of the included exercise intervention studies excluded participants with psychiatric comorbidities such as eating disorders and did not report other psychiatric comorbidity status, resulting in a lack of direct evidence regarding the moderating effect of this variable. Future studies should systematically assess psychiatric comorbidity status during recruitment and data analysis to examine its modifying effect on the exercise–food reward relationship.

#### 4.2.3 Analysis of the moderating effects of study design-related factors

At the study design level, factors such as the timing of food reward assessment, concomitant medication use, and the food environment may also influence the effects of exercise interventions on food reward.

Regarding the timing of food reward assessment, this review explored three dimensions: testing timing (pre-meal vs. post-meal), the interval between meal completion and testing, and the interval between exercise cessation and testing. With respect to pre-meal versus post-meal testing after exercise, we found that the proportion of intervention arms showing an inhibitory effect was higher under post-meal testing conditions than under pre-meal testing conditions, suggesting that the inhibitory effect of exercise on food reward may be more readily detectable in the postprandial state. This trend may arise because homeostatic satiety signals (e.g., the secretion of anorexigenic gastrointestinal hormones) act synergistically with exercise-induced reward suppression, jointly strengthening central inhibitory control over food cues. In contrast, in the pre-meal fasted state, hunger-driven reward motivation may mask the modulatory effects of exercise on reward circuitry (78). Regarding the interval between meal completion and testing, the proportion of time points at which a significant inhibitory effect was detected was higher for tests conducted within 30 min post-meal than for those conducted more than 5 h post-meal, indicating that the short postprandial window may be more sensitive for capturing reward-related changes. This can be attributed to the temporal overlap between meal-induced homeostatic inhibitory signals and exercise-mediated reward suppression, making the additional inhibitory effect of exercise more detectable during this period. Regarding the interval between exercise cessation and testing, a relatively higher proportion of assessments conducted >30 and ≤60 min post-exercise observed an inhibitory effect, suggesting a relatively concentrated time window for the effect of exercise on food reward. The underlying mechanism may be that, within a relatively short period, exercise-induced physiological changes (e.g., sympathetic activation, elevated levels of gut hormones such as PYY and glucagon-like peptide-1) have not yet fully returned to baseline. This state may temporarily reduce the reward valuation of energy-dense foods, particularly high-fat foods (79, 80). As the interval lengthens, these physiological signals gradually subside, and the reward system may return to baseline or even exhibit a compensatory response. It should be emphasized that the above stratified analyses are based solely on a semi-quantitative summary of the distribution of positive intervention arms, and no statistical comparisons of between-group effect sizes were conducted. The number of included time points in each subgroup was limited, and multiple temporal variables were intercorrelated, making it difficult to isolate the specific contribution of any single temporal parameter. Future original trials should standardize the reporting of meal status, postprandial waiting duration, and the interval between exercise cessation and testing, thereby harmonizing the assessment of temporal conditions and further elucidating the moderating role of assessment timing in exercise-induced modulation of food reward.

Pharmacological agents may exert synergistic or antagonistic effects on the outcomes of exercise interventions on food reward, either by directly acting on the central reward system or by modulating peripheral metabolic signals. Among these, weight-loss medications represented by GLP-1 receptor agonists can directly act on the mesolimbic reward circuitry, modulating the activity of GLP-1 receptors in the hypothalamus and mesolimbic reward pathways, thereby influencing food reward processing (81, 82). Randomized controlled trials by Farr et al. (83) and Ten Kulve et al. (84) have demonstrated that liraglutide, as one of the representative GLP-1 receptor agonists, significantly attenuates neural activation in the parietal cortex, insula, and putamen in response to high-reward food images. Selective serotonin reuptake inhibitors (SSRIs) represent another class of drugs known to modulate food reward processing, primarily by enhancing central serotonergic signaling. McCabe et al. (85) found that citalopram, a prototypical SSRI, significantly reduced neural activation in the ventral striatum and ventromedial/orbitofrontal cortex in response to chocolate reward stimuli, confirming that SSRI treatment can attenuate neural processing of rewarding stimuli. Taken together, the above evidence indicates that different pharmacological agents may modulate food reward processing through distinct neuropharmacological pathways. However, the potential moderating effects of these agents on exercise intervention outcomes remain largely uninvestigated. Theoretically, if exercise and a pharmacological agent modulate the reward system in the same direction, additive effects may emerge; conversely, if the agent blunts the plasticity of reward circuitry, the modulatory effects of exercise may be masked. It should be noted, however, that the vast majority of the included exercise intervention studies excluded participants who were taking medications that may affect appetite or body weight in order to control for this confounding factor. While methodologically justified, this design choice has resulted in a lack of direct evidence on exercise–medication interactions, precluding the evaluation of how medication status may modify the effects of exercise interventions. The moderating effect of pharmacological treatment on the exercise–food reward relationship therefore remains unknown. Future studies should consider including individuals on stable medication regimens for subgroup analyses or *post hoc* exploratory analyses, in order to preliminarily characterize the interaction patterns between pharmacological agents and exercise.

The food environment is a key external factor that modulates food reward processing, operating both through the sustained shaping of the reward system by everyday obesogenic environments and through the constraints that laboratory testing contexts impose on effect detection. With respect to everyday obesogenic environments, visual and multisensory cues associated with high-calorie foods are core drivers that upregulate food reward sensitivity. Studies have shown that brief, incidental exposure to advertisements or displays of high-fat, high-sugar foods can enhance implicit approach biases toward palatable foods in the general population, and such implicit reward associations can persist for at least 24 h (86). When multisensory stimulation is further superimposed, reward responses may become even stronger; related research has demonstrated that the simultaneous presentation of food images and odors significantly elevates reward-related physiological and subjective indicators such as salivation and eating urges (87). Long-term exposure to highly palatable food environments can reshape the baseline sensitivity of the mesolimbic dopamine reward circuitry, leading individuals to develop stable preferences for high-calorie foods. Against this background, environmental cues can override the exercise-induced suppression of food reward even when the body is in a state of negative energy balance, representing a significant external factor that interferes with the effectiveness of exercise interventions for weight management (88).

Beyond everyday obesogenic environments, differences in the design of laboratory testing contexts also markedly influence the detection of exercise intervention effects. In studies by Miguet et al. (7) and Fillon et al. (8), participants completed a buffet meal (allowing free choice of food type and portion size) before undergoing LFPQ-based food reward assessment, and significant post-exercise changes in food reward were observed in both studies. In contrast, Siroux et al. (10) employed a fixed meal design and did not detect significant alterations in food reward. This discrepancy may stem from the fact that the buffet paradigm provides a variety of foods and multisensory stimulation, thereby enhancing the incentive salience of food cues and amplifying the top-down inhibitory effect of exercise on the reward circuitry; conversely, the fixed meal setting, with its limited food selection, maintains the reward system at a lower level of baseline activation, making exercise effects more difficult to detect. It should be noted that the present review did not systematically quantify the relationships among the dose of food environment exposure, the duration of cue exposure, and food reward indicators, and the comparison between buffet and fixed meal designs was based on only a limited number of studies. Therefore, the current conclusions should be generalized with caution, and future research should systematically manipulate the above variables to further clarify the moderating role of the food environment.

## 5 Conclusions, limitations, and future directions

Existing evidence indicates that the modulation of food reward by exercise in individuals with obesity is characterized by both effect complexity and mechanistic systematicity. At the effect level, exercise intervention represents a potential means to regulate food reward behavior in individuals with obesity, with its effects moderated by multiple factors across three domains: (1) exercise intervention protocol-related factors, including exercise modality, intervention duration, frequency, session duration, exercise intensity, and energy expenditure (energy balance); (2) individual characteristics-related factors, including sex, age, body composition, metabolic health status, baseline physical activity level, habitual dietary patterns, exercise experience, and psychiatric comorbidities; and (3) study design-related factors, including the timing of food reward assessment, concomitant pharmacological treatment, and the food environment. At the mechanistic level, exercise intervention can achieve synergistic regulation through integrated neural-physiological-psychological multisystem pathways. In terms of cognitive-neural mechanisms, exercise can modulate the mesolimbic DA pathway, regulate EOS/ECS function, and restore the function of brain regions including the PFC and hippocampus, thereby achieving the integration of reward-related neural information. In terms of physiological-endocrine mechanisms, exercise can regulate gastrointestinal hormones and gut microbiota function, alleviate chronic low-grade inflammation, and ameliorate central insulin and leptin resistance, restoring the normal regulation of reward circuitry by metabolic signals. In terms of psychological-behavioral mechanisms, exercise can enhance inhibitory control, modulate attentional bias toward food cues, and improve emotion regulation, thereby attenuating the psychological drive for hedonic eating.

While systematically elucidating the modulatory effects of exercise on food reward in individuals with obesity, this review also reveals core limitations that urgently need to be addressed. First, the heterogeneity and inconsistency of exercise intervention effects are prominent, with similar exercise protocols even inducing opposite changes in food craving across different studies, suggesting that the individualized moderating variables influencing the exercise–reward relationship have yet to be effectively identified and integrated. Second, the key pathways through which exercise regulates the reward system have not been fully elucidated; although existing studies suggest that exercise may influence reward processing by improving inflammatory status and modulating the gut microbiota, direct causal evidence linking these pathways to improvements in reward behavior remains lacking. Third, the scientific basis for exercise prescription is weak; although current research has confirmed the moderating roles of factors such as intensity and type, the construction of personalized exercise prescriptions based on the core components of food reward (“liking” and “wanting”) still lacks a theoretical framework and empirical foundation. Fourth, the available evidence predominantly focuses on aerobic exercise and interval training, whereas intervention studies on resistance exercise remain extremely limited, constraining head-to-head comparisons of effects between different exercise modalities and the analysis of mechanistic differences. Fifth, the analysis of moderating variables is primarily based on semi-quantitative synthesis through subgroup stratification and qualitative discussion, lacking rigorous quantitative testing; moreover, the limited sample sizes within certain subgroups and the presence of confounding among variables to varying degrees mean that the observed moderating trends should be interpreted as exploratory findings. Sixth, the included primary studies commonly present inherent limitations such as inadequate reporting of randomization details and the intrinsic difficulty of blinding participants in exercise interventions, which introduced some degree of risk of bias. This reduces the overall strength of the evidence, weakening the robustness of the conclusions, and constraining the external validity of the findings.

Future research should strive to promote a scientific paradigm shift from population-level effects to individualized responsiveness in exercise interventions. Specifically, Future research should strive to promote a scientific paradigm shift from population-level effects to individualized responsiveness in exercise interventions. Specifically, First, systematically compare the differential effects of exercise parameters, individual baseline characteristics, and study design factors on various dimensions of food reward, evaluate the moderating effects of these variables, and identify the core sources of effect heterogeneity. Second, integrate multimodal behavioral, physiological, and neuroimaging data to elucidate the neural, physiological, and psychological mechanisms through which exercise modulates food reward. Third, develop a personalized exercise parameter matching system based on the “liking” and “wanting” reward phenotypes to inform precision exercise prescription. Fourth, conduct head-to-head comparisons of resistance exercise with aerobic and combined exercise in populations with obesity, incorporating measurements of myokines and skeletal muscle endocrine function to clarify the modality-specific modulatory effects of different exercise modalities. Fifth, strictly adhere to clinical trial reporting standards, fully disclose key methodological details such as randomization and allocation concealment, and implement outcome assessor blinding whenever feasible to reduce methodological bias and enhance the strength of evidence.

## Data Availability

Publicly available datasets were analyzed in this study. This data can be found here: No original raw data was generated in this narrative review. All analyses and conclusions are based on previously published peer-reviewed literature, which can be accessed via public academic databases including PubMed, Web of Science, Scopus and CNKI. Therefore, no data repository or accession number is available for this manuscript.
