# Correction: Exercise intervention modulates food reward in obesity: efficacy, mechanisms, and prospects

**DOI:** 10.3389/fnut.2026.2007059

**Published:** 2026-10-29

**Authors:** Dongfei Liu, Yucheng Jiang, Zhanfeng Wang, Xiaohe Mo

**Affiliations:** 1 School of Physical Education, Yunnan Normal University, Kunming, China; 2 School of Physical Education, Shihezi University, Shihezi, China; 3 Department of Scientific Research, Henan Sport University, Zhengzhou, China; 4 School of Physical Education, Yunnan Minzu University, Kunming, China

**Keywords:** exercise, food reward, intervention efficacy, mechanisms, obesity

There was a mistake in **2 Methods**, *2.1 Literature Search Strategy*, page 2, paragraph 2 as published.

The Figure 1, titled “Search strategy for PubMed”, was omitted in the published version.

**Figure 1:**
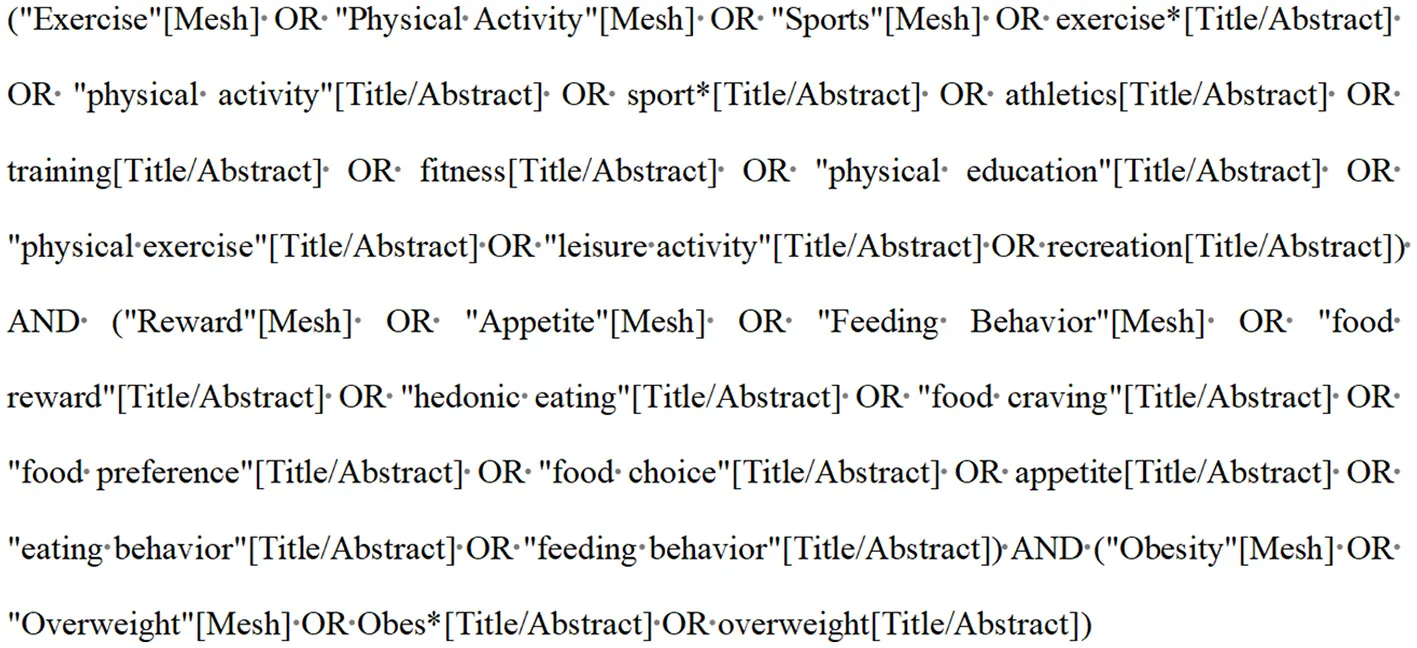
Search strategy for PubMed.

The figure has now been placed after the sentence “Taking PubMed as an example, the complete search string is as follows:”

The correct Figure appears below. The number of each subsequent figure has been updated accordingly.

The original version of this article has been updated.

